# Correlation of Shear Bond Strength and Degree of Conversion in Conventional and Self-Adhesive Systems Used in Orthodontic Bonding Procedures

**DOI:** 10.3390/biomedicines11051252

**Published:** 2023-04-23

**Authors:** Vjera Perković, Marina Šimunović Aničić, Vanni Lughi, Lucia Pozzan, Senka Meštrović, Gianluca Turco

**Affiliations:** 1 Department of Orthodontics, Faculty of Dental Medicine, University of Rijeka, 51000 Rijeka, Croatia; 2 Department of Orthodontics, School of Dental Medicine, University of Zagreb, 10000 Zagreb, Croatia; 3 Department of Engineering and Architecture, University of Trieste, 34127 Trieste, Italy; 4 Department of Medical Sciences, University of Trieste, 34125 Trieste, Italy

**Keywords:** adhesive dentistry, shear bond strength, degree of conversion

## Abstract

(1) Background: Self-adhesive systems have been proposed for the orthodontic bonding with the intention to reduce the traditional three-component system. (2) Methods: The sample consisted of 32 extracted intact permanent premolars randomly divided into two groups (*n* = 16). In Group I the metal brackets were bonded with Transbond XT Primer and Transbond XT Paste. In Group II the metal brackets were bonded with GC Ortho connect. The resin was polymerized for 20 s from two directions (mesial and occlusal) using a Bluephase light-curing unit. The shear bond strength (SBS) was measured using a universal testing machine. Immediately after SBS testing, Raman microspectrometry was performed for each sample to calculate the degree of conversion (DC). (3) Results: There was no statistically significant difference in the SBS between the two groups. A significantly higher DC (*p* < 0.001) value was recorded in Group II, in which the brackets were bonded with GC. Very weak or no correlation (0.01) was recorded between SBS and DC in Group I and moderate positive correlation was recorded in Group II (0.33). (4) Conclusions: No difference was found in SBS between the conventional and two-step systems used in orthodontics. The two-step system demonstrated higher DC compared to the conventional system. There is a very weak or moderate correlation between DC and SBS.

## 1. Introduction

Bond failure of the orthodontic brackets is a frequent event in daily clinical practice. Studies have demonstrated that bond failure occurs in 2.5–6.5% of the cases [1,2,3]. Since bond failure causes the orthodontic treatment to take longer than expected and increases operating costs, it is an inconvenient event, both for the practitioner and the patient.

Since the use of phosphoric acid etching for the bonding of orthodontic brackets was first documented by Newman in 1965 [4], different materials and protocols have been proposed for orthodontic bonding with the aim of gaining optimal bonding strength, reducing bonding time, and protecting the enamel surface. Usually, orthodontic adhesives are either composite resin or glass ionomer cement-based materials. The use of glass ionomer cements and resin-modified glass ionomer cements has many advantages because of the high fluoride content which can help to reduce enamel demineralization and caries development and does not demand moisture control. However, studies have shown that glass ionomer cements are associated with a significantly lower bond strength than composite resin [5,6], which makes composite resin commonly used material in orthodontic practice. Chemical-curing bonding systems were first used for orthodontic bonding. Their primary drawbacks were the long curing process and potential saliva contamination. The use of light-cured polymers for orthodontic bonding was initially explained by Tavas and Watts [7]. Orthodontic brackets are bonded to the enamel using three different substances in conventional composite adhesive systems: enamel conditioner, primer solution, and adhesive resin. The most traditional technique consists of three steps: phosphoric etching, resin layering, and composite paste bonding; it is still considered the gold standard. Recently, with the intention to reduce the traditional three-component bonding, two-step bonding has been introduced. Self-etch adhesives combine conditioning and priming in one step and self-adhesive systems combine primer and paste in one phase. Self-adhesive bonding materials do not require additional primer to adhere to the tooth surface. A reduction in clinical steps can be considered an important progression as it reduces operating time as well as possible mistakes, which can occur between the steps. Recent studies showed that a reduction in steps in orthodontic bonding made the chair time significantly faster [8,9,10]. However, self-adhesive systems, manufactured for restorative dentistry and prosthodontics, so far demonstrated lower shear bond strength (SBS) values in comparison with the traditional systems [11]. Their use for orthodontic purposes was not recommended as they showed poor bonding strength [12]. New self-adhesive systems have been introduced specifically for orthodontic bonding procedures such as GC Ortho connect. The bonding protocol in orthodontics includes several technique-sensitive steps and failures with composite resins are mostly attributed to moisture contamination [5]. Self-adhesive bonding materials in orthodontics are considered desirable materials, and their clinical application can be an important factor where moisture control and isolation are difficult to obtain.

The SBS values that have been reported as “clinically acceptable” are 6–8 MPa [13]. The SBS value in vivo should be enough to counteract the intraoral forces during the treatment as well as to allow the debonding process, while avoiding enamel damage. Different factors have been reported to have an influence on SBS such as the design of the bracket base pad, duration of acid etching, light-curing source, properties of the bonding material, and other factors [14].

The number of monomers that react to create polymers, or the proportion of C=C double bonds that are converted into C–C single bonds, is known as the degree of conversion (DC). The DC is a parameter of paramount importance in the light-curing procedure, as it shows the percentage of resin material that has been polymerized [15]. Orthodontic forces can be applied only after a successful polymerization. After the light polymerization procedure, the DC increases with time, mostly during the first day [16]. The DC for light cured polymers used in orthodontics depends on the monomer chemical structure, filler size, lighting source, time of curing, translucency, polymerization conditions, and concentration of the photoinitiator [17].

A larger DC leads to better physical properties of the material such as microhardness, resistance to wear, bond strength, and lower degradation over time [15]. Higher values of the DC indicate a reduction in double bonds, which is considered an advantage. Past studies have demonstrated that uncured monomer can leach from the polymerized resin and can cause undesirable effects such as allergic reactions and toxicity [17,18,19]. However, only a few studies investigated the correlation between the SBS and DC in materials used for orthodontic bonding [20,21]. Their findings demonstrated moderate or no positive correlation.

Therefore, the objective of the present study was to compare the SBS values, the ARI (adhesive remnant index) score, and the DC between two different bonding protocols applied in orthodontic practice, i.e., using conventional bonding material and self-adhesive bonding material, respectively—as well as to investigate the correlation between SBS and DC in each material tested.

To our knowledge, no correlation between the SBS and DC of the conventional bonding system and self-adhesive bonding system has been investigated yet. The hypothesis proposed for this study was that there is positive correlation between the SBS and the DC in both tested bonding systems, i.e., the conventional three components bonding system and the self-adhesive system.

## 2. Materials and Methods

### 2.1. Sample Size

The power analysis was performed using G*Power software (version 3.1.9.2, Franz Faul Universitat, Kiel, Germany). Based on the previous research [20], a total sample size of 32 was calculated with a power of 95% (actual power 9.5556), in order to disclose a significant difference with 0.66 effective size at the α = 0.05 significance level.

### 2.2. Sample Preparation

A total number of 32 recently extracted permanent premolars (first and second, both upper and lower premolars) were collected, cleansed with water, and placed in saline solution for no longer than three months. The teeth were intact, meaning that the inclusion criteria were permanent premolars with no cavities, no white spot lesions, and no dental fillings or other restorations. Teeth had been extracted for reasons unrelated to the objectives of this study, mostly due to the orthodontic treatment requirements. Written informed permission was given and signed by each patient. The teeth were randomly assigned into two groups (*n* = 16), Group I and Group II.

### 2.3. Tooth Mounting

Prior to bonding, cold-curing methacrylate resin was used to set the teeth in cubic plastic molds (Orthocryl, Dentaurum GmbH & Co. KG, Ispringen, Germany). Using a mounting jig, the buccal surface of each tooth was oriented perpendicularly to the bottom of the mold. After a period of one hour, the teeth were removed from the molds and returned to the saline solution until the bonding procedure.

### 2.4. Bonding Procedure

The orthodontic bonding materials used in this study are shown in Table 1. In Group I, the conventional material (three-step procedure) was used, and the self-adhesive material (two-step procedure) was used in Group II.

Stainless-steel premolar brackets (American Orthodontics, Sheboygan, WI, USA) were bonded in the center of each tooth’s buccal surface so that the longitudinal axis of the bracket was parallel to the tooth’s longitudinal axis. The metal brackets’ bases had a rectangular form and a fine mesh adhesive surface, which could be easily fitted onto the buccal curvature of the premolars. Prior to the light-curing procedure any excess bonding material was removed with a probe.

In Group I (conventional, three-step system), the teeth were etched using 37% phosphoric acid (Ultra-Etch, Ultradent Products Inc., South Jordan, UT, USA) that was applied for 15 s, rinsed with water for 20 s, and then air-dried for 10 s. The bracket bonding was performed using Transbond XT Primer and Transbond XT Paste (3M Unitek, Monrovia, CA, USA).

In Group II (two-step self-adhesive system), the teeth were etched using 37% phosphoric acid (Ultra-Etch, Ultradent Products Inc., South Jordan, UT, USA) that was applied for 15 s, rinsed with water for 20 s, and then air-dried for 10 s. The bracket bonding was performed using GC Ortho connect (GC America, Alsip, IL, USA).

The bonding material was polymerized for 20 s from two directions (mesial and occlusal related to the tooth) using a Bluephase light-curing unit (Ivoclar, Vivadent, Schaan, Liechtenstein) with a power output of 1100 mW/cm^2^. The results were recorded using a Bluephase meter (Ivoclar, Vivadent, Schaan, Liechtenstein), which was checked before every bonding. The same operator prepared all the specimens (V.P.).

### 2.5. Shear Bond Strength Testing (SBS)

**Figure 1:**
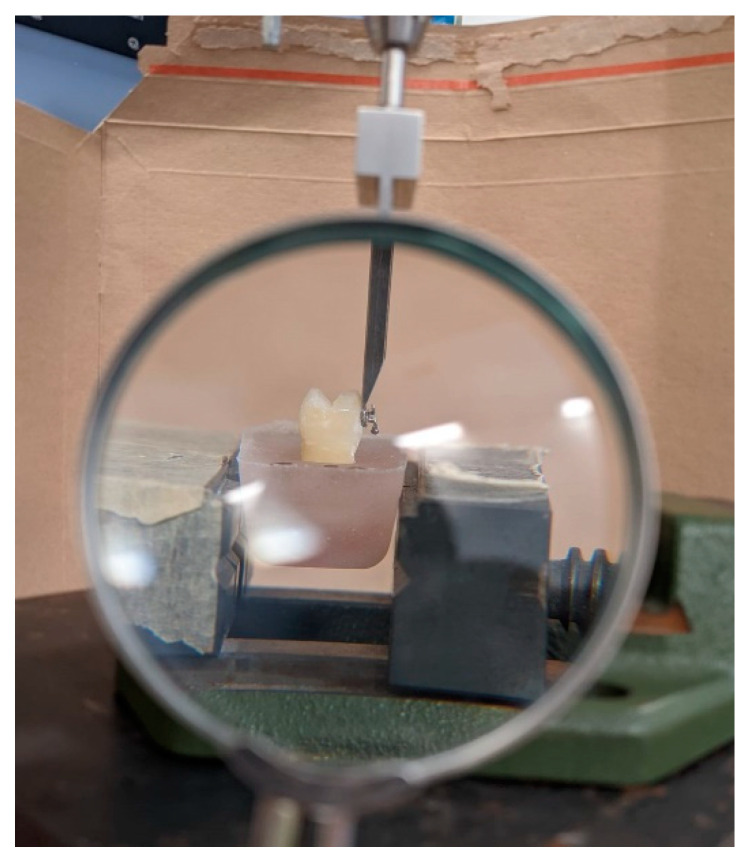
SBS testing with stationary magnifier.

Using a universal testing machine (AGS-X 10, Shimadzu Corporation, Kyoto, Japan), SBS testing was performed. A chisel-shaped loading device was used to apply a shear force with a crosshead speed of 1 mm/min parallel to the bracket’s surface. To make sure that the force was applied parallel to the surface, a stationary magnifier was used (Figure 1).

The highest shear force needed to debond each bracket was noted as a force (N) and was later converted into SBS (MPa) by dividing the force value with the surface area of the bracket’s base. The manufacturer (American Orthodontics, Sheboygan, WI, USA) specified that the surface area of the bracket base was 10.83 mm^2^.

### 2.6. Adhesive Remnant Index (ARI)

A stereomicroscope (Nikon SMZ645, Nikon, Tokyo, Japan) with a 10× magnification was used to observe the area after debonding and establish the adhesive remnant index (ARI) [22], which has its range from 0 to 3. The scores can be explained as follow: 0 = no resin left on the surface of the enamel, 1 = less than half of the resin, 2 = half or more of the resin, and 3 = all the resin with a clear impression of the bracket mesh.

### 2.7. Degree of Conversion (DC)

Immediately after the SBS test, Raman microspectrometry was performed using a Renishaw InVia Raman Microscope (Renishaw plc., Gloucestershire, UK) and was completed within thirty minutes after light initiation with the purpose of minimizing any possible dark-cure effects. Three separate locations were defined on each of the bracket’s base, one in the center and one on each side of the bracket (mesial and distal side). The locations were marked “Near”, “Center”, and “Far”, based on the distance from the light-curing unit, with “Near” being the closest. If there was enough material on the base, Raman microspectrometry was performed on the base. In case there was no material on the bracket, Raman microspectrometry was carried out on the enamel surface of the specimen.

**Figure 2:**
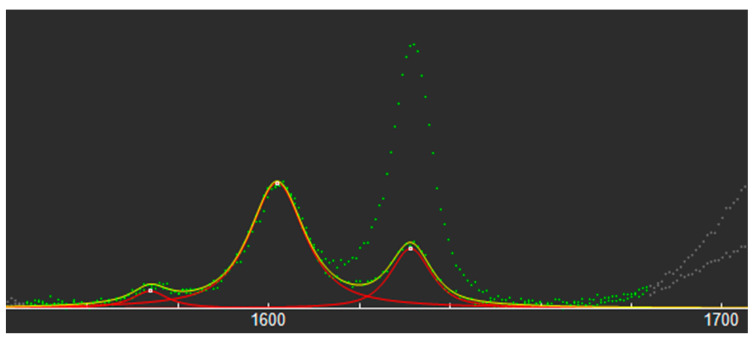
Raman spectra for polymerized and unpolymerized GC Ortho Connect. Green line represents experimental data acquired from Raman microspectrometry; Red line represents three bell-shaped functions; Yellow line represents fitting curve; Gray line represents deselected data.

Raman spectra were preliminarily collected from the resin before and after polymerization, in order to identify the most suitable spectral features for quantifying the DC. The spectra were nearly identical, except for the relative intensities of some of the Raman bands (Figure 2).

The relative intensities of the bands at 1632 cm^−1^ and at 1602 cm^−1^ were found to be particularly sensitive to the polymerization, as well as rather isolated from the neighboring ones—thus, enabling an easier quantitative analysis. DC was calculated as follows:

(1) $$\mathrm{DC}\left(\%\right)=100\times \left(1-\frac{R_{POLYMERIZED}}{R_{UNPOLYMERIZED}}\right)$$

where *R* = band area at 1632 cm^−1^/band area at 1602 cm^−1^.

### 2.8. Data Analyses

Data were analyzed using the SPSS statistical software (IBM SPSS Statistics version 21 for Mac, Chicago, IL, USA). Within each Group, SBS and DC were evaluated quantitatively and were analyzed using descriptive statistics such as mean, standard deviation, and range. DC was calculated in the three points identified above. According to the Kolmogorov–Smirnov Test, the data from the SBS, DC, and adhesive remnant index testing were normally distributed. A *t*-test (*t*-test, α = 0.05) was performed to compare the bond strength and the DC between the groups.

To determine if the correlation between SBS and DC exists, in each group as well as in three different points, the Pearson correlations were used.

## 3. Results

### 3.1. SBS

The mean SBS value for the group with the three-step bonding procedure was 14.82 MPa, while in the two-step group it was 13.71 MPa, but no statistically significant differences were observed between the groups in terms of the SBS values (Table 2).

### 3.2. ARI Scores

After the debonding procedure, the debonded area was examined using a stereomicroscope. In both groups, most of the examined surfaces had an ARI score of 1 (15 in group I and 11 in group II) and no statistically significant differences were observed between them (Table 3).

### 3.3. DC

A significantly higher DC (*p* < 0.001) value was recorded in Group II, in which the brackets were bonded using the two-step bonding protocol (Table 4).

### 3.4. Correalation

Very weak or no correlation (0.01) was recorded between SBS and DC in Group I and a moderate positive correlation was recorded in Group II (0.33).

### 3.5. DC in Three Points

In Group I (Transbond XT), all three points where the DC was measured showed weak or no correlation with SBS (Table 5).

In Group II (GC) points “Near” and “Far” showed a moderate positive correlation with SBS, 0.41 and 0.34, respectively. The “Center” point showed a weak positive correlation (0.21) (Table 5).

Group II had higher DC values than Group I in all three key points of the bracket area (Table 5 and Table 6). In group II, the “Center” point had the highest mean value of the DC (75%).

## 4. Discussion

The focus of this research was to determine whether the reduction of clinical steps in orthodontic bonding, from conventional three-step (Group I, Transbond XT) to two-step self-adhesive (Group II, GC), has any effect on the SBS, adhesive remnant index, and DC’s results, as well as to investigate the correlation between SBS and DC in two bonding materials.

The crosshead speed of 1 mm/min in SBS testing, used in this study, has been used in many relevant studies [23,24,25]. Additionally, the literature shows that the crosshead speed between 0.5 and 10 mm/min has no significant impact on the SBS [26]. The SBS values obtained in this study can be considered clinically sufficient as Reynolds et al. [13] proposed 6–8 MPa for minimal clinical values. Although, it must be taken into consideration that this is an in vitro study and comparisons with in vivo should be done with caution. Secondarily, in comparison with other research, no thermocycling or any kind of aging of the sample was carried out as the correlation between the SBS and the DC was the main objective. The SBS and the DC testing was performed immediately after bonding to diminish any dark-cure effect on the SBS or the DC.

Our results show higher SBS values than reported in previous research. However, the comparison between the SBS values from different studies should be done with caution as different methodologies have been applied. Moreover, water storage decreases bond strength on average by 10.7 MPa as shown in the systematic review [27]. In general, the interpretation of the SBS values obtained in vitro as clinically acceptable is imprudent.

In this research, the two-step system refers to eliminating the use of the primer, usually applied after etching and before the application of composite paste, and it was shown that this two-step bonding system can be a good alternative to the conventional three-step system (etch, primer, and bond). However, different research showed different results. Asiri et al. [28] concluded that, when reducing the steps of bonding procedures, composite bond strength was reduced as well. To be noted, the research used self-etching flowable composite, meaning, it eliminated etching as a clinical step, and resulted in lower SBS of the bonding material when bonded to the dentin. Another study investigated SBS in the two-step system compared to the conventional system (Transbond XT) and showed that, even though SBS in the self-etching system was lower than in the conventional system, it was still within the range that is clinically acceptable [29]. Another study evaluated a novel epoxy-based resin material for orthodontic bonding, again with reduced clinical steps (using self-etching primer). It showed very low levels of SBS, even less than is clinically acceptable, which made it inappropriate for orthodontic use [30].

Transbond XT adhesive and paste are one of the standard materials used for bonding in orthodontics and has been the focus of many past studies investigating the SBS [31,32,33,34,35]. In a recent study, the same two-step system was used for bracket bonding and the results in the control group (where Transbond XT was used) showed similar SBS values as those obtained in the present study [36]. We found only one study [37] investigating the self-adhesive system, and it does not show significant difference in the SBS with respect to Transbond XT, which is in agreement with our results.

Although bonding is an important procedure, debonding is also crucial in order to accomplish successful orthodontic treatment. Debonding aims to remove the brackets and adhesive while returning the enamel surface as closely as possible to its pretreatment state. The adhesive remnant index is one of the most frequently used methods to assess how much adhesive is still adhered to the teeth following bracket removal. The ARI score does not dependent on the SBS as much as on the design of the bracket base and properties of the bonding agent [38]. No significant differences between the two groups in ARI score were revealed. Most values were 1, which implied a superior occurrence of bond failure at the enamel–adhesive interface. The ARI scores for the self-adhesive group are in accordance with previous studies [36,37,39,40,41].

To minimize any dark-cure effect, within a period of up to 30 min after the light initiation, the Raman microspectroscopy was carried out to determine the DC. This method was chosen over Fourier transform infrared (FTIR) spectroscopy because it was proven to be more adequate for determining the DC [42,43]. As demonstrated in the previous research [44,45], to calculate the DC we used the double bonds of the methacrylate group at 1632 cm^−1^ and the aromatic double bond at 1602 cm^−1^.

Results for the DC of Transbond XT showed comparable values with the recent study [46] and higher values compared to the other study [16], although some differences in methodology should be accounted for.

To the best of our knowledge, there was no study that investigated the DC in a self-adhesive system (GC ortho connect). In this study, the results demonstrated significantly higher DC in self-adhesive system compared to the three-step one. A possible explanation for this result can be seen in the composition of the self-adhesive system as it has lower viscosity. A recent study, investigating different orthodontic resins, showed that there is a relationship between the viscosity of the material and its DC, as the low viscosity resins tend to have higher DC [47]. Although, minimal values for the DC in clinical orthodontics have not been established yet; in restorative resins the range for the DC is 55–75% [48]. Both results for the DC in this study are in this range. In this study, the DC was measured in three places under the bracket’s base, which contributes to greater clinical significance, in contraposition to many studies where brackets were not used at all. The DC is shown to have similar values in all three points measured in both materials. Interestingly, this points out that polymerization is equally efficient both closer to and further from the light-curing unit. This result provides us with an important clinical implication, i.e., that the exact positioning of the light-curing unit is not crucial in the polymerization process.

A moderate positive correlation between the SBS and the DC was found in the self-adhesive group. In the literature, we could not find any other study investigating the correlation of the self-adhesive system used in this study. No correlation was found in Group I (conventional system) and this agrees with recent investigations [21]. A recent study [49] concluded, based on their results, that the SBS did not depend on the DC. When the correlation was checked for each point, only the self-adhesive material showed a moderate positive correlation to SBS in points “Near” and “Far”, with “Near” showing the highest correlation. Further research in this field is required, possibly with a larger sample to enable more general conclusions. In vivo studies are needed as well, to investigate results within a clinical environment.

## 5. Conclusions

Based on this research we can conclude the following:

- No difference was found in SBS values between the conventional and self-adhesive systems used in orthodontics. The self-adhesive systems can be used as a good alternative as they reduce clinical steps.
- The self-adhesive system demonstrated a higher DC compared to the conventional system.
- No significant difference was found in the adhesive remnant index score between the two materials.
- There is an overall very weak or moderate correlation between the DC and SBS values in the self-adhesive system. The DC has minimal effect on the SBS.
- The polymerization was equally effective both in the points closer to and further from the light-curing unit.

## Figures and Tables

**Table 1:** Materials used in present study.

Name	Composition	Manufacturer
**Transbond XT primer**	Bis-GMA, TEGDMA, dimethylamono-bezenethanol, DL-camphorquinone, hydrochinone	3M Unitek, Monrovia, CA, USA
**Transbond XT Light Cure Adhesive**	Silane-treated quartz, Bis-GMA, Bis-EMA, silane-treated silica, diphenyliodonium hexa-fluorophosphate	3M Unitek, Monrovia, CA, USA
**GC Ortho Connect**	7.7.9-trimethyl-4.13-dioxo-3.14-dioxa-5.12-diazahexadecane-1.16-diyl bismethacrylate	GC America, Alsip, Illinois, USA

Bis-GMA: bisphenol-A-glycidyldimethacrylate; TEG-DMA: triethyleneglycol dimethacrylate; Bis-EMA: ethoxylated bisphenol-A-dimethacrylate.

**Table 2:** Descriptive statistics and SBS comparison of two groups.

Group	Number	SBS				
		Mean	Standard Deviation	Minimum	Maximum	Significance *t*-Test
**I**	16	14.82	2.12	10.17	18.94	*p* = 0.308
**II**	16	13.71	3.71	7.85	19.34	

**Table 3:** Fracture modes after SBS testing.

Group	Total Number of Cases	ARI				Significance Mann Whitney U Test
		Score 0 (Number of Cases)	Score 1 (Number of Cases)	Score 2 (Number of Cases)	Score 3 (Number of Cases)	
**I**	16	0	15	1	0	*p* = 0.968
**II**	16	2	11	2	1	

**Table 4:** Descriptive statistics and DC (%) comparison of two groups.

Group	Number	SBS				
		Mean	Standard Deviation	Minimum	Maximum	Significance *t*-Test
**I**	16	62.94	3.46	58.18	71.06	*p* = 0.0000113 *p* < 0.01
**II**	16	73.68	7.08	52.54	83.32	

**Table 5:** Descriptive statistics and DC (%) for each of the three points measured in Group I and correlation with SBS comparison of two groups.

Group I	Mean	Minimum	Maximum	SD	R
DC “Center”	62.95	57.02	72.36	0.04	0.01
DC “Near”	65.30	55.85	96.65	0.09	0.1
DC “Far”	62.27	52.65	70.81	0.05	0.02

**Table 6:** Descriptive statistics and DC (%) for each of three points measured in Group II and correlation with SBS comparison of two groups.

Group II	Mean	Minimum	Maximum	SD	R
DC “Center”	74.50	54.78	85.54	0.07	0.21
DC “Near”	70.71	51.84	82.38	0.08	0.41
DC “Far”	71.55	14.24	80.82	0.15	0.34

## Data Availability

Data is contained within the article.

## References

[1] Aguirre M.J., King G.J., Waldron J.M. (1982). Assessment of bracket placement and bond strength when comparing direct bonding to indirect bonding techniques. Am. J. Orthod..

[2] Thiyagarajah S., Spary D.J., Rock W.P. (2006). A clinical comparison of bracket bond failures in association with direct and indirect bonding. J. Orthod..

[3] Read M.J.F., O’Brien K.D. (1990). A clinical trial of an indirect bonding technique with a visible light-cured adhesive. Am. J. Orthod. Dentofac. Orthop..

[4] (1965). Newman George V Epoxy adhesives for orthodontic attachments: Progress report. Am. J. Orthod..

[5] Costa A.R., Correr A.B., Puppin-Rontani R.M., Vedovello S.A., Valdrighi H.C., Correr-Sobrinho L., Vedovello Filho M. (2012). Effect of bonding material, etching time and silane on the bond strength of metallic orthodontic brackets to ceramic. Braz. Dent. J..

[6] Bishara S.E., Vonwald L., Olsen M.E. (1999). Effect of time on the shear bond strength of glass ionomer and. Am. J. Orthod. Dentofac. Orthop..

[7] Tavas M.A., Watts D.C. (1979). Bonding of orthodontic brackets by transillumination of a light activated composite: An in vitro study. Br. J. Orthod..

[8] Aljubouri Y.D., Millett D.T., Gilmour W.H. (2004). Six and 12 months’ evaluation of a self-etching primer versus two-stage etch and prime for orthodontic bonding: A randomized clinical trial. Eur. J. Orthod..

[9] Banks P., Thiruvenkatachari B. (2007). Long-term clinical evaluation of bracket failure with a self-etching primer: A randomized controlled trial. J. Orthod..

[10] Elekdag-Turk S., Isci D., Turk T., Cakmak F. (2008). Six-month bracket failure rate evaluation of a self-etching primer. Eur. J. Orthod..

[11] Al-Saleh M., El-Mowafy O. (2010). Bond strength of orthodontic brackets with new self-adhesive resin cements. Am. J. Orthod. Dentofac. Orthop..

[12] Chu C.H., Ou K.L., Dong D.R., Huang H.M., Tsai H.H., Wang W.N. (2011). Orthodontic bonding with self-etching primer and self-adhesive systems. Eur. J. Orthod..

[13] Reynolds I.R., von Fraunhofer J.A. (1976). Direct Bonding of Orthodontic Brackets—A comparative study of adhesives. Br. J. Orthod..

[14] Al-Hity R., Gustin M.P., Bridel N., Morgon L., Grosgogeat B. (2012). In vitro orthodontic bracket bonding to porcelain. Eur. J. Orthod..

[15] Rahiotis C. (2010). Degree of cure and monomer leaching from orthodontic adhesive resins: In vitro and in vivo evidence. Semin. Orthod..

[16] Wichai W., Nimcharoensuk K., Anuwongnukroh N., Dechkunakorn S., Roongrujimek P. (2018). Degree of conversion of three light-cured orthodontic adhesives. Key Eng. Mater..

[17] Çörekçi B., Malkoç S., Öztürk B., Gündüz B., Toy E. (2011). Polymerization capacity of orthodontic composites analyzed by Fourier transform infrared spectroscopy. Am. J. Orthod. Dentofac. Orthop..

[18] Anne Rathbun M., Craig R.G., Hanks C.T., Filisko F.E. (1991). Cytotoxicity of a BIS-GMA dental composite before and after leaching in organic solvents. J. Biomed. Mater. Res..

[19] Thompson L.R., Miller E.G., Bowles W.H. (1982). Materials Science: Leaching of Unpolymerized Materials from Orthodontic Bonding Resin. J. Dent. Res..

[20] Henbest N. (2013). Orthodontic Bracket Bond Strength and Resin Composite Adhesive.

[21] Ponikvar M.J. (2014). Effect of Delayed Polymerization Time and Bracket Manipulation on Orthodontic Bracket Bonding. ProQuest Diss. Master Thesis.

[22] Årtun J., Bergland S. (1984). Clinical trials with crystal growth conditioning as an alternative to acid-etch enamel pretreatment. Am. J. Orthod..

[23] Jia L., Stawarczyk B., Schmidlin P.R., Attin T., Wiegand A. (2012). Effect of caries infiltrant application on shear bond strength of different adhesive systems to sound and demineralized enamel. J. Adhes. Dent..

[24] Mews L., Kern M., Ciesielski R., Fischer-Brandies H., Koos B. (2015). Shear bond strength of orthodontic brackets to enamel after application of a caries infiltrant. Angle Orthod..

[25] Gaur A., Maheshwari S., Verma S., Tariq M. (2016). Effects of adhesion promoter on orthodontic bonding in fluorosed teeth: A scanning electron microscopy study. J. Orthod. Sci..

[26] Yamaguchi K., Miyazaki M., Takamizawa T., Tsubota K., Rikuta A. (2006). Influence of crosshead speed on micro-tensile bond strength of two-step adhesive systems. Dent. Mater..

[27] Finnema K.J., Özcan M., Post W.J., Ren Y., Dijkstra P.U. (2010). In-vitro orthodontic bond strength testing: A systematic review and meta-analysis. Am. J. Orthod. Dentofac. Orthop..

[28] Asiri A.A., Khan R., Alzahrani S.S., Haider S., Khan S.U. (2021). Comparative Analysis of the Shear Bond Strength of Flowable. Coatings.

[29] Bhattacharjee D., Sharma K., Sahu R., Neha K., Kumari A., Rai A. (2021). Comparative Evaluation of Shear Bond Strength of Brackets Bonded with self Etch Primer/Adhesive and Conventional Etch/Primer and Adhesive System. J. Pharm. Bioallied Sci..

[30] Brauchli L., Steineck M. (2021). Siloranes–suitability of a novel adhesive for orthodontic bracket bonding. Dent. J..

[31] Naidu E., Stawarczyk B., Tawakoli P.N., Attin R., Attin T., Wiegand A. (2013). Shear bond strength of orthodontic resins after caries infiltrant preconditioning. Angle Orthod..

[32] Ekizer A., Zorba Y.O., Uysal T., Ayrikcil S. (2012). Effects of demineralizaton-inhibition procedures on the bond strength of brackets bonded to demineralized enamel surface. Korean J. Orthod..

[33] Costenoble A., Vennat E., Attal J.P., Dursun E. (2016). Bond strength and interfacial morphology of orthodontic brackets bonded to eroded enamel treated with calcium silicate-sodium phosphate salts or resin infiltration. Angle Orthod..

[34] VelI I., Akin M., Baka Z.M., Uysal T. (2015). Effects of different pre-treatment methods on the shear bond strength of orthodontic brackets to demineralized enamel. Acta Odontol. Scand..

[35] Anicic M.S., Goracci C., Juloski J., Miletic I., Mestrovic S. (2020). The influence of resin infiltration pretreatment on orthodontic bonding to demineralized human enamel. Appl. Sci..

[36] Khalil A.S., Tamish N.M., Elkalza A.R. (2022). Assessment of chemical, ultrasonic, diode laser, and Er:YAG laser application on debonding of ceramic brackets. BMC Oral Health.

[37] Iglesias A., Flores T., Moyano J., Artés M., Gil F.J., Puigdollers A. (2020). In vitro study of shear bond strength in direct and indirect bonding with three types of adhesive systems. Materials.

[38] O’Brien K.D., Watts D.C., Read M.J.F. (1988). Residual debris and bond strength-Is there a relationship?. Am. J. Orthod. Dentofac. Orthop..

[39] Bishara S.E., VonWald L., Laffoon J.F., Warren J.J. (2001). Effect of a self-etch primer/adhesive on the shear bond strength of orthodontic brackets. Am. J. Orthod. Dentofac. Orthop..

[40] Flores T., Mayoral J.R., Giner L., Puigdollers A. (2015). Comparison of enamel-bracket bond strength using direct- and indirect-bonding techniques with a self-etching ion releasing S-PRG filler. Dent. Mater. J..

[41] Bishara S.E., VonWald L., Laffoon J.F., Jakobsen J.R. (2000). Effect of altering the type of enamel conditioner on the shear bond strength of a resin-reinforced glass ionomer adhesive. Am. J. Orthod. Dentofac. Orthop..

[42] Pianelli C., Devaux J., Bebelman S., Leloup G. (1999). The micro-Raman spectroscopy, a useful tool to determine the degree of conversion of light-activated composite resins. J. Biomed. Mater. Res..

[43] Gilchrist F., Santini A., Harley K., Deery C. (2007). The use of micro-Raman spectroscopy to differentiate between sound and eroded primary enamel. Int. J. Paediatr. Dent..

[44] Imazato S., McCabe J.F., Tarumi H., Ehara A., Ebisu S. (2001). Degree of conversion of composites measured by DTA and FTIR. Dent. Mater..

[45] Iliadi A., Baumgartner S., Athanasiou A.E., Eliades T., Eliades G. (2014). Effect of intraoral aging on the setting status of resin composite and glass ionomer orthodontic adhesives. Am. J. Orthod. Dentofac. Orthop..

[46] de Sena L.M.F., Barbosa H.A.M., Caldas S.G.F.R., Ozcan M., Souza R.O.d.A.E. (2018). Effect of different bonding protocols on degree of monomer conversion and bond strength between orthodontic brackets and enamel. Braz. Oral Res..

[47] de Araujo L.O.F., Barreto O., de Mendonça A.A.M., França R. (2015). Assessment of the degree of conversion in light-curing orthodontic resins with various viscosities. Appl. Adhes. Sci..

[48] Ferracane J.L., Greener E.H. (1986). The effect of resin formulation on the degree of conversion and mechanical properties of dental restorative resins. J. Biomed. Mater. Res..

[49] Yılmaz B., Bakkal M., Zengin Kurt B. (2020). Structural and mechanical analysis of three orthodontic adhesive composites cured with different light units. J. Appl. Biomater. Funct. Mater..

